# Effect of cycle ergometer and conventional exercises on rehabilitation of older patients with total hip arthroplasty: study protocol for randomized controlled trial

**DOI:** 10.1186/s13063-015-0647-8

**Published:** 2015-04-08

**Authors:** Mariana Kátia Rampazo-Lacativa, Maria José D’Elboux

**Affiliations:** Nursing Faculty, State University of Campinas (UNICAMP), Postgraduate in Health Sciences, Campinas, SP Brazil; Associate Professor of Nursing Faculty, State University of Campinas (UNICAMP), Campinas, SP Brazil

**Keywords:** Hip arthroplasty, Aged, Exercise therapy, Rehabilitation, Cycle ergometer

## Abstract

**Background:**

Total hip arthroplasty (THA) is an increasingly common treatment for older patients with hip osteoarthritis. The best strategy for a physiotherapy intervention for older people after THA is not clear in the literature. The purpose of this protocol study is to test the feasibility of undertaking a full trial clinical to evaluate the effect of ergometer cycling-associated conventional exercises on functional results and health-related quality of life (HRQOL) of older patients with THA.

**Methods/Design:**

This study protocol is a prospective, single center, randomized controlled pilot clinical trial. Older patients (≥60 years) in the postoperative phase after primary unilateral THA for hip osteoarthritis will be consecutively recruited for this study and randomly allocated to 2 treatment groups. Group I will perform cycle ergometer and conventional exercises, and group II will perform only conventional exercises. The sessions will be conducted twice a week for 8 weeks. Assessments will be made at baseline (2 weeks postoperatively: the moment that the patients receive a referral for physical therapy, which will start after suture removal), after intervention (10 weeks postoperatively), and at 6 months of follow-up (24 weeks postoperatively). The primary outcomes are the function, evaluated using the Harris Hip Score (HHS) and the Short Physical Performance Battery (SPPB). The secondary outcome is HRQOL, measured using 2 evaluation instruments: the Medical Outcomes Study 36-Item Short-Form Health Survey (SF-36) and the Western Ontario and McMaster Universities Osteoarthritis Index (WOMAC). Data collectors will be blinded and will not have contact with participants during the interventions.

**Discussion:**

This randomized controlled trial will provide evidence regarding the effect of this exercise therapy on physical function and quality of life in older patients after THA. If our hypothesis is correct, both interventions will be effective, but the exercises on the cycle ergometer conferring better results in function, physical performance and quality of life. The study follows Consolidated Standards of Reporting Trials (CONSORT) guidelines, and the approval of the local ethics committee has been obtained.

**Trial registration:**

ClinicalTrials.gov: NCT01622465 (14 June 2012)

## Background

Total hip arthroplasty (THA) is one of the most frequently performed orthopedic surgeries. Its benefits have been widely documented; in particular, improvements in pain and function in patients with severe hip osteoarthritis (OA) [1-3]. With the worldwide phenomenon of a growing older population, this surgical procedure is being performed more frequently and has consequently increased the number of older patients in rehabilitation [4]. In this context, it becomes necessary to know how this profile of patients responds to the various resources available for rehabilitation in the postoperative period, since the best strategy for a physiotherapy intervention is not clear in the literature [5,6].

After THA, deficits in muscle strength and the limitations on physical function, developed during the evolution of OA, may still be present for many months [7]. Functional deficits in older patients with THA deserve greater attention because they can significantly influence quality of life [8]. The objective of rehabilitation is guided by the early restoration of functional capacity, and this age group, with the physical changes of aging itself, requires a longer time to achieve the desired functional levels [9]. Furthermore, the rehabilitation process also includes the prevention, minimization, and restoration of possible psychological, emotional, and social impacts arising from, and concomitant with, the functional deficits of these patients.

Clinical trials have been proposed to investigate different strategies of rehabilitation after THA [6]. However, few have been conducted exclusively with older patients who have specific physical, psychological, and social characteristics.

Reduced pain and stiffness and increased function have been demonstrated in older patients who received hydrotherapy after THA [10]. The effect of muscle electrical stimulation on quadriceps and triceps surae muscles was investigated in another study of older patients with THA. The addition of this resource to conventional exercise was effective in the recovery of muscle strength of the knee extensors and functional independence of these patients [11]. The inclusion of the ergometer arm in a conventional exercise program also improved pain, stiffness, and function in older patients undergoing THA [12].

In a study by Liebs *et al*. [13], a group of adult and older patients with THA performed cycle ergometer exercises in addition to conventional exercises in the early postoperative period and experienced significant improvements in health-related quality of life (HRQOL) and satisfaction with results of surgery. However, more information is needed to determine the effect of this resource, which is available in most rehabilitation centers, on functional outcomes and quality of life in older patients with THA.

### Objectives and hypotheses

The aims of this prospective, single center, randomized controlled pilot study are:

- to test the feasibility of undertaking a full trial clinical;
- to identify if the cycle ergometer will be well tolerated by older patients with THA in the early phase of rehabilitation;
- to test the feasibility of the proposed outcome measures;
- to identify effect size at end of treatment in order to calculate an appropriate sample size for the full trial clinical.

## Methods/Design

### Study design

The study is a prospective, parallel-group, single center, randomized pilot study conducted in accordance with Consolidated Standards of Reporting Trials (CONSORT) guidelines [14].

### Participants and recruitment procedures

Patients aged 60 years and older who undergo THA at the Clinical Hospital of the State University of Campinas, Brazil, and are recommended to participate in rehabilitation in the physical therapy and occupational therapy service of that institution will be consecutively recruited for this study. Inclusion and exclusion criteria are listed in Table 1.

**Table 1:** **Inclusion and exclusion criteria for participation in the study**

**Inclusion criteria**	**Exclusion criteria**
Sixty years old and older	Hip fracture
Diagnosis of osteoarthritis	Postoperative complications: dislocation, infection, cardiovascular
Primary unilateral total hip replacement	Revision arthroplasty
No physical therapy performed within 2 months prior to surgery	Neuromuscular disease that compromises motor function
	Unable to attend the physical therapy sessions at the study institution
	Refusal to participate

Initial contact will occur after the surgery and before discharge, when information about the study will be provided to patients. At that time, eligible patients will be identified and invited to participate, and will receive verbal and written information about the trial (background, procedure, and randomization). The patients will receive a consent form and a referral for physical therapy, which will start after suture removal (2 weeks postoperatively). After obtaining informed consent from each participant the baseline information will be collected at the time of the first physical therapy session. After the intervention (10 weeks postoperatively) and at 6-month follow-up after the surgery (24 weeks postoperatively) the patients will be assessed and the analysis outcomes will be performed.

**Figure 1:**
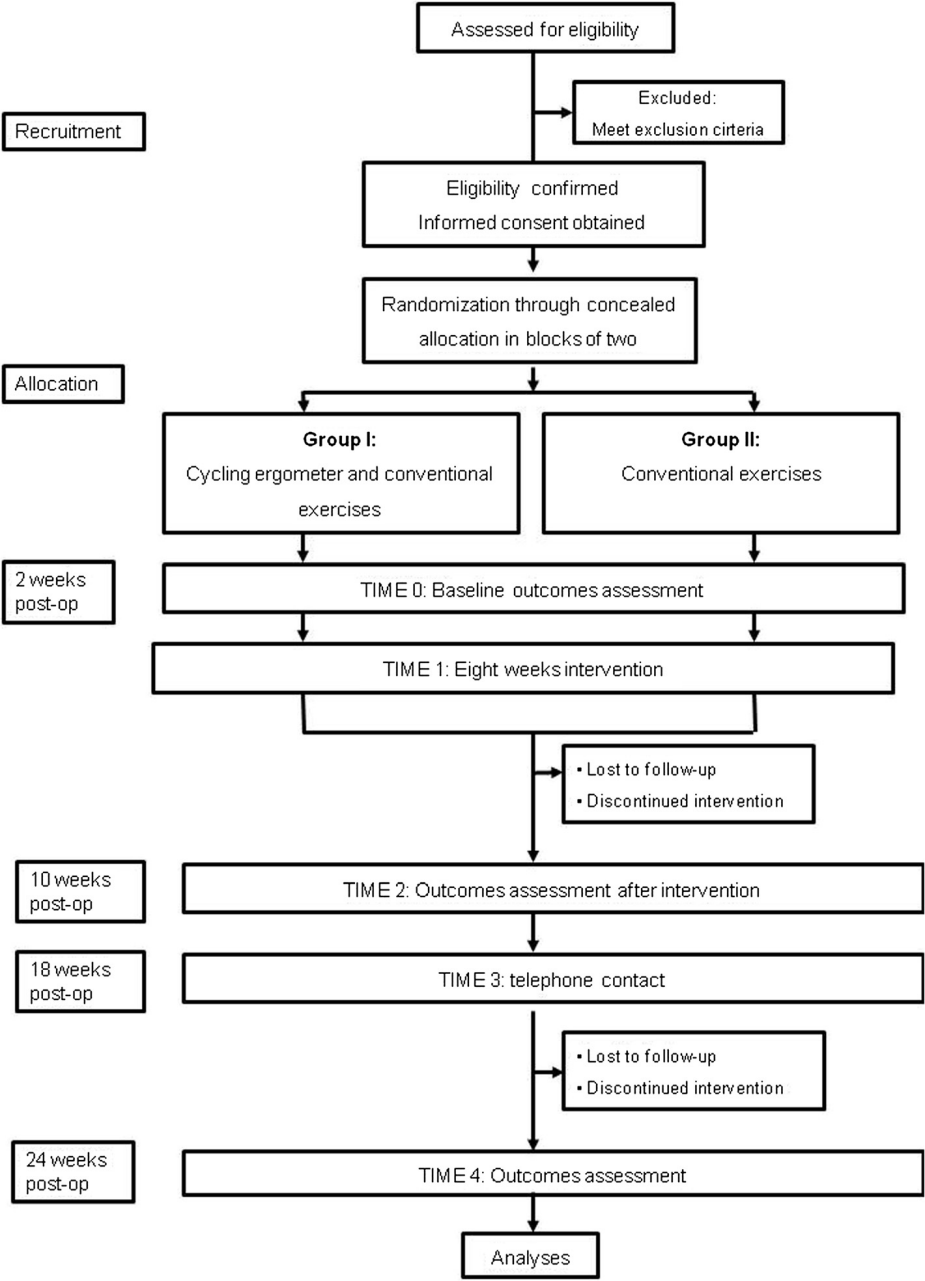
**Flow diagram of the randomized clinical trial.**

A flow diagram of the trial’s progression (recruitment, randomization, intervention allocation, follow-up, and data analysis) is shown in Figure 1.

### Randomization and allocation concealment

A randomization sequence will be created using a computer-generated list of random numbers in block sizes of two. The randomization will be computer generated using Proc Plan, SAS® software Version 9.1.3 (SAS Institute Inc., Cary, NC, USA). Patients will be allocated to one of two interventions: cycle ergometer and conventional exercises (group I) or conventional exercises alone (group II). All participants will have the same probability of inclusion in either group. The allocation sequence will be concealed in sequentially numbered opaque, sealed envelopes. The envelopes with the random sequence will be in the possession of a person not involved in the trial. Envelopes will be opened only after the enrolled patients have completed all baseline assessments.

### Blinding

Because of the nature of the interventions, patients and intervention applicator (principal investigator) will not be blinded. However, there will be blinding of data collectors responsible for baseline and follow-up assessments, and these physiotherapists will be trained to apply the assessment tools used to collect data for statistical analysis and interpretation of outcomes. For blinding to be maintained the data collectors will not have contact with participants during the intervention and the follow-up. The contact is only at the time of evaluation outcomes measures. The physiotherapy sessions will be conducted individually, in schedules that do not coincide with the presence of the physiotherapist data collector or other participant.

### Interventions

All subjects will receive the same clinical and orthopedic care during hospitalization; that is guidelines for positioning in bed, transfers, limitations of range of motion (ROM) (hip flexion ≤ 90 degrees and hip adduction only to midline) and adaptations in the home (raising the heights of the toilet, bed, sofa, and chairs if necessary).

All subjects will receive a conventional-exercises program. Conventional-exercise sessions will last 50 minutes and be performed 2 days a week for 8 weeks. At the end of the intervention, patients will have performed 16 sessions and will be 10 weeks postoperative.

**Figure 2:**
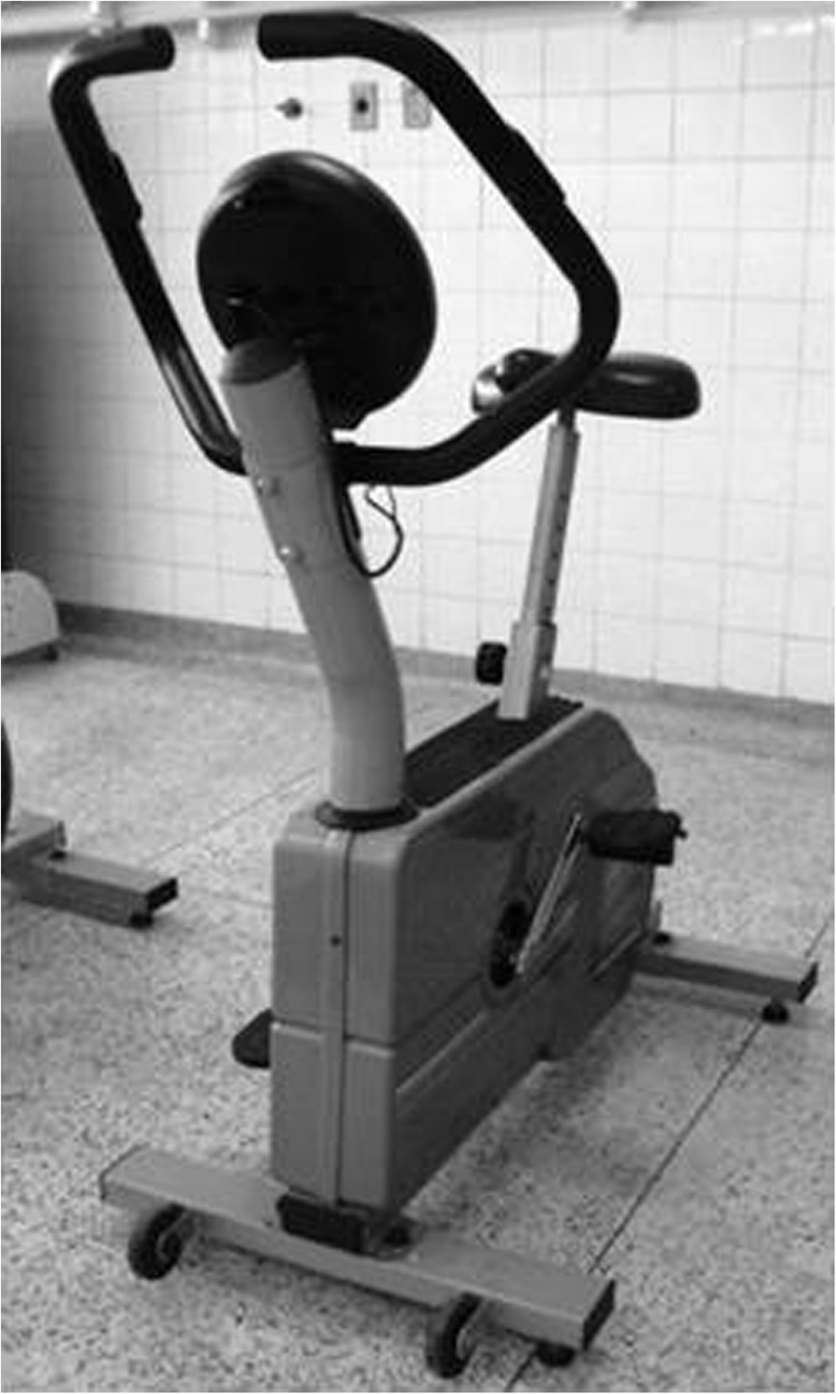
**Photograph of the cycle ergometer.**

Under the guidance of a physical therapist, group I will perform cycle ergometer exercises on a vertical, stationary cycle ergometer, Dyamond line, Uniforce Fitness, Dy, model EB 497E (contato@uniforcefitness.com.br) (Figure 2) and conventional exercises and group II will perform conventional exercises alone. Ergometer resistance will be minimal (30 W) because the aim is not to perform cardiac exercise. The duration of ergometer cycling will be increased gradually, starting with 10 minutes in the first 2 sessions and increasing to 20 minutes for the remainder, and its aims are to improve coordination, proprioception, ROM, and muscle strength. For this reason, changes are not made during performance of the exercise; frequency is maintained at 60 revolutions per minute and the resistance is also maintained at the starting level. The height of the saddle will be set so that the forefoot will reach the pedal with the knee in minimum flexion (5 to 10 degrees). Special attention will be given to monitoring heart rate and blood pressure before, during, and after exercise. Patients will be instructed to follow the exercises on the cycle ergometer under the supervision of a physiotherapist, ensuring the execution of this only in the presence of the professional.

Conventional exercises will consist of exercises to improve joint ROM, muscle strength, function, balance, coordination and gait. A description of conventional exercises is provided in Table 2.

**Table 2:** **Description of conventional exercises used in total hip arthroplasty rehabilitation**

**Weeks postoperative**	**Lower-limb weight-bearing/Assistive device for walking**	**Description**
2 to 4	Partial/Walker	• Stretching (30 seconds each muscle group): hip flexors, extensors, adductors; knee flexors, extensors; ankle dorsiflexors and plantar flexors
		• Active assisted range of motion (5 to 10 repetitions): hip flexion, extension, abduction, external rotation; knee flexion, extension
		• Muscle strengthening (1 to 3 sets of 10 to 15 repetitions):
		o Hip flexors, extensors, abductors (low-resistance rubber band fixed to the ankle in standing position)
		o Knee extensors (sandbag fixed to the ankle in a seated position)
		• Transfer training: supine to side - lying in bed; sit and stand bed and chair
		• Gait training using a parallel bar and assistive device
4 to 6	Total/Crutch or cane	• Stretching maintained
		• Active range of motion (5 to10 repetitions): hip flexion, extension, abduction, external rotation; knee flexion, extension
		• Muscle strengthening (1 to 3 sets of 10 to 15 repetitions):
		o hip flexors (moderate-resistance rubber band fixed to the ankle in standing position)
		o hip abductors (standing position and seated position with moderate-resistance rubber band)
		o hip extensors (bridge exercise, one- and two-footed in supine position)
		o knee extensors and flexors (weight-training equipment, in a seated position, 20% 1RM)
		• Balance and gait training: walking on an unstable surface, backward walking, side step with the use of parallel bars if necessary
6 to 8	Total/With or without cane	• Stretching maintained
		• Muscle strengthening (1 to 3 sets of 10 to 15 repetitions):
		o hip flexors and abductors maintained
		o hip extensors (bridge exercise, one- and two-footed in supine position with ball)
		• Climbing and descending stairs
		• Coordination, balance, and gait training: circuits with stairs, obstacles, changing direction, changing speed
8 to 10	Total	• All exercises maintained
		• Addition of the muscle strengthening of hip and knee extensors with squat exercises (1 to 3 sets of 10 to 15 repetitions)

RM, repetition maximum.

Description of conventional exercises used in total hip arthroplasty rehabilitation.

### Intervention and follow-up periods

Assessments will be made at baseline (2 weeks postoperatively), after intervention (10 weeks postoperatively) and at 6-month follow-up (24 weeks postoperatively), and both treatment groups will have completed the intervention after 10 weeks. After the 10 weeks of supervised intervention, patients will be advised to perform some exercises unsupervised at home. In the period between the end of supervised intervention and the final evaluation (at 24 weeks), patients will be able to telephone the physiotherapist for assistance, and there will be a phone contact in the 18th week postoperatively to assess the patients' health condition.

### Outcome measures

At baseline, an instrument for sociodemographic and clinical characterization will be used to obtain data regarding age, sex, race, marital status, education, weight, height, body mass index, comorbidities, medications, joint pain, and lifestyle. Data regarding THA surgery outcome, the primary clinical outcome, will be the mean change in the hip function and physical performance of the lower limbs. The instruments used will be: Harris Hip Score (HHS) [15] and the Short Physical Performance Battery (SPPB) [16]. The mean change in total score in these instruments between the 2 arms, as measured at baseline, 10 and postoperative weeks postoperative will be the primary outcome measure. We will also collect: reason for surgery, waiting time to perform the surgery prosthesis fixation, affected side, hospital stay, postoperative complications, availability of a caregiver after surgery, adaptations at home, and satisfaction with the results of surgery and rehabilitation.

### Primary outcome measures

The feasibility outcome will include an evaluation of eligibility, recruitment and retention rates (in line with CONSORT recommendations), as well as monitoring of completion of outcome measures and assessments. The primary clinical outcome will be the mean change in the hip function and physical performance of the lower limbs. The instruments used will be: the HHS [15] and the SPPB [16]. The mean change in total score in these instruments between the 2 arms, as measured at baseline, 10 and 24 postoperative weeks will be the primary outcome measure.

The HHS questionnaire, an instrument that was developed to evaluate the results of THA, has been validated abroad [17] and in Brazil [18]. It consists of a scale ranging from 0 to 100 points in 4 domains: pain, function, deformity, and ROM. The maximum scores are 44 points for the pain domain and 47 for the functional domain, the latter being subdivided into activities of daily living (14 points) and gait (33 points). Domains are scored based on subjects’ responses obtained through interviews, with the exception of the deformity and ROM domains, which are evaluated by the examiner with the use of a tape measure and goniometer. The deformity domain can be scored from 0 to 4 and the ROM domain from 0 to 5, with higher scores indicating greater ROM, but within of limits for prosthesis, and less deformity. It is considered a poor functional outcome if the HHS total score is less than 70 points, fair if the score is between 70 and 79; good if the score is between 80 and 89, and excellent for 90 to 100 points [15,18].

The SPPB comprises tasks that assess balance, gait speed, and lower-limb strength in older people. It was developed in 1994 by Guralnik *et al*., with support from the National Institute on Aging, for the Established Populations for Epidemiologic Studies of the Elderly project in the US [16]. It is an effective tool for evaluating the physical performance of the lower limbs in the older population and consists of three tests that evaluate, in sequence, static standing balance; gait speed at a normal pace; and, indirectly, muscle strength in the lower limbs through the motion of getting up from a chair and sitting down again five consecutive times without the aid of the upper limbs. Each test is scored on a scale ranging from 0 (poor performance) to 4 points (optimal performance). The SPPB total score is obtained by adding the scores for each test and ranges from 0 (poor performance) to 12 points (best performance) [16,19]. The results may be interpreted as inability or very poor performance (0 to 3 points), low performance (4 to 6 points), moderate performance (7 to 9 points) and high performance (10 to 12 points) [20-22].

### Secondary outcome measures

HRQOL will be considered a secondary outcome and the questionnaires used to assess this will be the Medical Outcomes Study 36-Item Short-Form Health Survey (SF-36) [23] as a generic instrument and the Western Ontario and McMaster Universities Osteoarthritis Index (WOMAC) [24] as a specific instrument (measured at the same time points).

The SF-36 is a generic tool for assessing HRQOL that has been translated and validated in Brazil [25]. It consists of 36 items comprising 8 categories: functional capacity (10 items), physical aspects (4 items), pain (2 items), general health status (5 items), vitality (4 items), social aspects (2 items), mental health (5 items) and a question comparing current health conditions with those from a year ago. Each question is assigned a score and the scores tabulated and normalized to a scale of 0 to 100, where 0 corresponds to worst health status and 100 to best health status. There are no cutoff points, and each category is evaluated separately [23,25].

The WOMAC is a specific instrument that measures quality of life in patients with OA of the hip and knee [24]. Its use is indicated for postoperative evaluation of total arthroplasty of the knee (TKA) or hip [26]. The questionnaire was originally intended to be self-administered; however, it has been used for telephone interviews, and recently a computer touch-screen version has been validated. It comprises 24 items divided into 3 categories. The pain category has five questions, the joint stiffness category two questions, and the physical disability category seventeen questions. Each question has five possible answers using a Likert scale representing difficulty, and the responses *none* or *never*, *mild* or *monthly*, *moderate* or *weekly, severe* or *daily*, and *extreme* or *always* correspond to scores of 0, 1, 2, 3 and 4, respectively. Thus, 0 represents the absence of the symptom and 4 the worst manifestation of that symptom. Summing the scores, each category receives a score that is normalized to a scale of 0 to 100 points, with 0 representing the best health status and 100 the worst possible status [24,27].

### Feasibility outcomes

The feasibility outcome will include an evaluation of eligibility, recruitment and retention rates (in line with CONSORT recommendations), as well as monitoring of completion of outcome measures and assessments. Refusal, withdrawal and dropout from the study protocol will be recorded. Acceptability of the intervention using the cycle ergometer will be assessed by measuring the time to fully perform the exercise and by structured questionnaires with research participants on completion of the intervention. The feasibility of data collection strategies will be assessed descriptively and through item response rates and physical tests.

### Statistical analysis

To describe the sample profile descriptive statistics of numerical variables (scores of scales) will be made.

To compare numerical variables between the two groups at baseline we will use the Mann-Whitney test, due to lack of normal distribution of variables. To compare longitudinal measures between groups and times we will use analysis of variance (ANOVA) for repeated measures, followed by Tukey’s multiple comparison test to compare the groups at all times, and the profile test contrasts to analyze the evolution between assessments in each group. The variables will be transformed into posts (ranks) in the absence of normal distribution.

To estimate the sample size and the power of the sample we will use the sample size calculation for longitudinal studies with repeated measures to compare variables between the two groups (I and II) over the two collection times (pre and post intervention). The significance level will be defined (alpha or Type I error) at 5%, calculating the effect size or delta, which is the expected difference or difference obtained between the group means divided by the standard deviation, and the coefficient of intraclass correlation between repeated measurements. The values of the effect size delta (delta effect size) will vary generally between 0 and 3. Delta values of 0.25, 0.75 and 1.25 respectively correspond to small, medium or large effects. Subsequently, the power of the sample and the sample size will be obtained by setting the power at 80% or Type II error (1-beta) at 20%. The significance level for statistical tests will be 5% (*P* < 0.05).

Statistical analysis of the data will be performed using SAS® software, Version 9.1.3 for Windows (SAS Institute Inc., Cary, NC, USA).

### Ethics

The trial complies with the Declaration of Helsinki, and approval has been granted by the Ethics Committee of the Medical Sciences Faculty of the State University of Campinas: approval number 403/2011. The trial is registered at ClinicalTrials.gov (NCT01622465).

## Discussion

The cycle ergometer has been studied as a resource in the rehabilitation of patients with THA and TKA, but with a sample composed of both adults and older people. According to two systematic reviews on the topic, no clinical trials were found that investigated the effect of cycle ergometer-associated exercises with conventional exercises in a group composed exclusively of older patients in the postoperative period after THA [5,6].

When proposing exercise therapies for rehabilitation after THA, it should be considered that the aging process itself brings significant biological, psychological, and social decline. Decreased muscle strength and, depending on other health conditions, serious functional decline can occur [28]. Hip OA can also result in muscle imbalance, pain, and stiffness, which may further compromise function in older patients [29]. Accompanying these physical losses may be impacts on their psychological, emotional, and social aspects of quality of life [30,31].

In patients with severe hip OA, THA aims to relieve pain partially or completely and consequently allow improved function and quality of life [2]. Even considering the risks of a surgical procedure, such as infection, the benefits of THA, when properly indicated, have been demonstrated [1-3,5,6,17]. In this context, patients and professionals seek to achieve the best functional levels in the postoperative rehabilitation period. However, aspects of the rehabilitation of older patients should be considered [31]. The restoration of ROM and muscle strength may be slower because of reduced muscle elasticity and reduced ability to recruit muscle fibers [9,28].

Thus, the resources employed in physical therapy, which have been used and investigated frequently in adults, should be better understood when applied to older patients. The cycle ergometer activates the muscle groups of the hip, knee, and ankle; favors the increase and maintenance of joint ROM of the lower limbs and requires motor coordination for the execution of movements [32]. Liebs *et al*. [13] evaluated the effects of cycle ergometer-associated exercises in patients with THA and TKA in the early postoperative period. Patients performed cycle ergometer-associated exercises 3 times per week for 3 weeks. In patients with THA, pain and function, as assessed by the WOMAC, were found to be improved 3 months postoperatively in the group that performed cycle ergometer-associated exercises compared with patients who received only conventional physical therapy. No significant improvements were observed in patients with TKA.

The sessions will be supervised by the same physiotherapist and all exercises individualized, with progression guided by the patient’s function and pain level. Blood pressure and heart rate will be noted at the beginning and end of the session as well as before, during and after cycle ergometer-associated exercise.

Primary outcome measures include two instruments: the HHS for functional evaluation of the hip and the SPPB for evaluation of performance of the lower limbs. The inclusion of the HHS was justified by its frequent use in the local orthopedic community for functional hip assessments in addition to its recommendations in the literature and evidence of validity and reproducibility for patients undergoing THA [17]. These authors emphasize that this assessment system is the most widely used in the evaluation of hip arthroplasty [17]. The SPPB is an effective tool for evaluating the physical performance of the lower limbs in the older population and has been used in research on aging because of its high sensitivity in identifying changes in functionality [33,34].

The secondary outcome measures used in this trial are patient-reported outcome measures broadly applied in interventional and observational studies in patients with THA [8,10,12,13]. Recommendations that both generic and specific measures be included in the assessment of HRQOL can be found in the literature [35,36].

Some problems and limitations in conducting this trial may interfere with its effect. First, we emphasize that the participants in the intervention and the applicator will not be blinded. Second, participants in the control group, who will not perform cycle ergometer-associated exercises, will know when reading the consent form that this resource will not be used for their rehabilitation but will be used by other patients with the same condition. This may generate some disincentive; however, this feature will be offered to the control group after 10 weeks postoperatively.

Loss to follow-up may occur over the course of the study and may be considered a third limitation. The study will be conducted at a regional referral hospital where patients from neighboring cities are treated. Difficulties in transporting patients to physical therapy sessions can lead to missed sessions or the inability to continue rehabilitation at this institution. In these cases, referrals and guidance will be given for rehabilitation services in neighboring towns and patients will return to the study institution only for clinical follow-up.

Another consideration is that the exclusion criteria limit the generalization of the results to other populations. Even in the case of a pilot clinical trial, the results may not be generalized to patients with THA who are unable to perform moderate-intensity exercise, such as in cases of cardiovascular complications in the postoperative period.

This randomized clinical trial has been designed with the main purpose of evaluating the feasibility of undertaking a trial to compare the effect of cycle ergometer-associated conventional exercises to conventional exercises alone in older patients with THA. The results of the pilot trial will be used to inform the design of the future definitive study, which would provide evidence relating to the inclusion of this resource in the functional recovery of these patients. In accordance with CONSORT guidelines for reporting of clinical trials, the results will be submitted to a peer-reviewed international journal for publication irrespective of the outcome.

## Trial status

The trial began in August 2011. Participant recruitment will likely be completed after 2 years.

## Abbreviations

ANOVA: analysis of covariance
CONSORT: Consolidated Standards of Reporting Trials
HHS: Harris Hip Score
HRQOL: health-related quality of life
OA: osteoarthritis
ROM: range of motion
SAS: Statistical Analysis System
SF-36: Medical Outcomes Study 36-Item Short-Form Health Survey
SPPB: Short Physical Performance Battery
THA: total hip arthroplasty
TKA: total knee arthroplasty
WOMAC: Western Ontario and McMaster Universities Osteoarthritis Index.
